# Effect of yogic exercise on super oxide dismutase levels in diabetics

**DOI:** 10.4103/0973-6131.36792

**Published:** 2008

**Authors:** Hemant H Mahapure, Sanjay U Shete, T K Bera

**Affiliations:** Department of Scientific Research, Kaivalyadhma Yoga Institute, Lonavla, Maharashtra, India

**Keywords:** Diabetes, glycosylated hemoglobin, reactive oxygen species, superoxide dismutase, Yogic exercises

## Abstract

**Context:**

Reactive oxygen species are known to aggravate disease progression. To counteract their harmful effects, the body produces various antioxidant enzymes, *viz*, superoxide dismutase, glutathione reductase etc. Literature reviews revealed that exercises help to enhance antioxidant enzyme systems; hence, yogic exercises may be useful to combat various diseases.

**Aims:**

This study aims to record the efficacy of yoga on superoxide dismutase, glycosylated hemoglobin (Hb) and fasting blood glucose levels in diabetics.

**Settings and Design:**

Forty diabetics aged 40–55 years were assigned to experimental (30) and control (10) groups. The experimental subjects underwent a Yoga program comprising of various *Asanas* (isometric type exercises) and *Pranayamas* (breathing exercises) along with regular anti-diabetic therapy whereas the control group received anti-diabetic therapy only.

**Materials and Methods:**

Heparinized blood samples were used to determine erythrocyte superoxide dismutase (SOD) activity and glycosylated Hb levels and fasting blood specimens collected in fluoride Vacutainers were used for assessing blood glucose.

**Statistical Analysis Used:**

Data were analyzed by using 2 × 2 × 3 Factorial ANOVA followed by Scheffe's posthoc test.

**Results:**

The results revealed that Yogic exercise enhanced the levels of Superoxide dismutase and reduced glycosylated Hb and glucose levels in the experimental group as compared to the control group.

**Conclusion:**

The findings conclude that Yogic exercises have enhanced the antioxidant defence mechanism in diabetics by reducing oxidative stress.

A major internal threat to the cellular homeostasis of aerobic organisms arises from free radical intermediates and the byproducts generated from oxygen metabolism. The paradox of aerobic life is that aerobic organisms cannot exist without oxygen and yet oxygen happens to be inherently dangerous to their very existence. This is because ironically, these reactive oxygen species (ROS) are derived from normal physiological and metabolic processes that are essential to the living cell.[1] A free radical is any species that is capable of independent existence and contains one or more unpaired electrons.[2] Oxygen becomes eligible to be called a free radical as it contains two unpaired electrons, each in a different orbital and both spinning in the same direction. The unpaired electrons alter the chemical reactivity of an atom or molecule, more often rendering it more reactive than the corresponding non-radical.[3] The reductive environment of the cellular milieu provides ample opportunities for the oxygen to go through spontaneous univalent reduction. Thus, superoxide anion, hydrogen peroxide and the extremely reactive hydroxyl radicals are common products of life in an aerobic environment and these agents are responsible for oxygen toxicity.[3] To continue to exist in such an adverse ambience, living organisms generate a number of antioxidant compounds mostly comprising of a variety of antioxidant enzymes, viz, superoxide dismutase, glutathione peroxidase, glutathione reductase etc, whose key objective is to seize and inactivate the generated reactive oxygen species.[1,2]

Morbidity and mortality in diabetes is typically associated with the development of its various related complications like atherosclerosis, nephropathy and other microvascular complications. Oxidative stress as well as peroxidation of cellular structures, which is a consequence of increased free radical activity, are thought to play an important role in the accelerated degenerative changes in diabetes.[4–6] Oxidative stress as assessed by the index of lipid peroxidation has been shown to be elevated in diabetics.[7] In diabetes, excess oxygen radicals may result from the autooxidation of glucose,[8] although there is considerable evidence that antioxidant defence is depleted and that the activity of antioxidant enzymes is reduced.[9]

While ancient Indian culture has perceived and promoted the merits of yogic practices, researchers have been interested in finding out what actually results from various yogic practices. In addition, they have sought to discover a meeting ground on points of mutual interest so that medicine and yoga together can achieve optimum functioning of not only the body but also of the mind. It is believed that regular practice of yoga brings about a decrease in stress levels and improved antioxidant status.[10] The present study was carried out to assess the effect of yogic exercises on superoxide dismutase activity in diabetic subjects along with glycosylated Hb and blood glucose levels. This was done to get some idea about the antioxidant status of these subjects after specific yogic practices and to promote its significance and worth in controlling the disease's progress.

## SUBJECTS AND METHODS

### Subjects

The experimental group comprised of 21 male and nine female Type II Diabetes Mellitus patients in the age groups of 40–55 yrs and 40–50 yrs respectively on regular diet and anti-diabetic drug regimens. The subjects in this group underwent a Yoga program comprising of various *Asanas* (isometric type exercises) and *Pranayamas* (breathing exercises) along with the anti-diabetic therapy. The control group comprised of six male and four female Type II Diabetes Mellitus patients in the age groups of 40–58 yrs and 45–54 yrs, respectively with regular diet and anti-diabetic drug regimens. Parameters assessed were red blood cell (RBC, erythrocyte) Superoxide Dismutase, glycosylated Hb and fasting blood glucose levels.

#### Blood sample

Heparinized blood samples were used to determine erythrocyte superoxide dismutase (SOD) activity and glycosylated Hb level whereas fasting blood specimens collected in fluoride Vacutainers were used for assessing blood glucose.

#### Determination of SOD activity

Erythrocyte SOD was determined with a Randox test combination (Randox, Crumlin, U.K.). Xanthine and xanthine oxidase were used to generate superoxide radicals reacting with 2-(4-iodophenyl)-3-(4-nitrophenol)-5-phenyltetrazolium chloride (INT) to form a red formazan dye. Concentrations of the substrates were 0.05 mM for xanthine and 0.025 mM for INT. Superoxide dismutase inhibits this reaction by converting the superoxide radical to oxygen. One unit of SOD activity is considered to be that which inhibits the rate of reduction of INT by 50% in a complex system with xanthine and xanthine oxidase. Due to the small linearity range of the test, the sample was diluted so that the percentage of inhibition fell between 30 and 60%. A standard curve was prepared using the kit's standard and the value for the diluted sample was read from this curve. SOD activity was measured at 505 nm from hemolysates of washed erythrocytes obtained by centrifugation of whole blood. Results were expressed in U/ml.[11–14]

#### Determination of glycosylated hemoglobin

This was done by using a cation exchange resin chromatography method commercially available from Monozyme India Limited by Monozyme Glycohemin. A hemolysate was prepared by mixing the erythrocytes with the lysing reagent to eliminate schiff's base. The hemolysate is further subjected to form a mixture with cation exchange resin where the non-glycosylated fraction separates, rendering the glycosylated fraction free in the supernatant. This fraction is measured at 415 nm and is calculated by using the proportionate comparison with total Hb levels measured at 415 nm as the linearity range of the assay is quite high, *i.e*., 4.0 to 20%.[15]

#### Determination of blood glucose

Blood glucose was determined using the Glucose Oxidase-Peroxidase method available with Point Scientific Inc USA. The plasma glucose is converted to gluconic acid and hydrogen peroxide in the presence of glucose oxidase. This hydrogen peroxide is further degraded by peroxidase liberating nascent oxygen in the presence of phenol and 4-aminoantipyrine to form a pink quinine-imine complex that is measured at 546 nm.[16,17]

#### Prescribed Yogic exercises and their justification

A specific set of Yogic exercises was prescribed beginning with *Suryanamaskar* (dynamic stretching of the muscles of the abdomen, back, neck, hands and legs), *Shavasana* (muscle relaxation), *Tadasan* (leg muscles stretching), *Konasana* (twisting and stretch of the spine), *Pawanmuktasana* (hip and back muscles stretching), *Naukasana* (abdominal muscles stretch), *Bhujangasana* (exercises the lower back muscles), *Sarpasana* (exercises the lower back muscles by twisting), *Dhanurasana* (exercises the whole back and muscles), *Ardhamastyendrasana* (twisting the spine), *Paschimottanasana* (hamstring stretch), *Yogamudra* (pressure on the lower abdomen and back stretch), *Brahmamudra* (neck muscles stretch), *Anulom-Vilom* and *Ujjayi Pranayama* (breathing exercises) and *recitation of the word ‘OM’* respectively.[18] All of these Yogic exercises have been selected on the basis of the following mechanisms:

1. Yogic exercises have a direct influence on pancreatic secretion by rejuvenation of the pancreatic cells through alternate abdominal contractions and relaxation.[19]
2. Reduction in blood glucose levels due to muscular exercise involved in the asanas.[19]

It is to be noted that apart from the stated *Asanas* (postural exercises), special emphasis was been given on the breathing exercises, i.e., *Pranayama*. Also, meticulous care was taken during these breathing practices as affirmed in the yogic texts, i.e., the breathing is consciously made slow, deeper and rhythmic. This type of breathing can be gradually learned and further developed to produce precise physiological effects. One can acclimate and train the breathing organs to these specific patterns of breathing without much strain on other systems. *Pranayama* is done by deep inspiration followed by holding the breath and then ultimately, expiration (*according to the yogic texts, these are conventionally called Puraka/Kumbhaka/Rechaka phases*). The ratio of these was observed as 1:4:2 for each of the phases respectively.[20] The objective of this type of pranayama is to have a controlled breathing pattern and monitor the sustenance of physiology with a limited load of oxygen so as to observe its implication on the oxidative status. Other yogic practices mentioned were implemented to improve stress malleability, compliance and to achieve the stated objectives in diabetic subjects under study. The above practices were scheduled in the morning for an hour everyday consecutively for six weeks except on Sundays.

## RESULTS

The results of descriptive statistics evaluating the central tendency and dispersion [Table 1] of the selected variables revealed that the pretest mean scores of the control and experimental groups for SOD (Superoxide dismutase), glycosylated Hb and blood glucose were mostly similar. This would seem logical because the subjects of both the control and experimental groups were matched prior to experimental intervention. The post-test results of the experimental group [Table 1] indicate favorable changes in the means of almost all the selected variables whereas an opposite trend, although not statistically significant, is evident in the case of the control group.

**Table 1:** Mean (standard deviation, SD) of superoxide dismutase (SOD) activity and glycosylated Hb and blood glucose levels in diabetics

Variables	Cont. Gr.		Exp. Gr.	
		
	Pre	Post	Pre	Post
SOD (U/ml)	171.09 (4.65)	167.90 (3.47)	172.50 (3.82)	211.35 (4.88)
Glycosylated Hb (%)	10.39 (0.82)	10.36 (0.70)	10.35 (0.78)	(0.63) 9.01
Blood glucose (Fasting) mg%	200.11 (9.87)	192.26 (8.54)	189.54 (9.90)	162.38 (8.60)

However, the results of 2 × 2 × 3 Factorial ANOVA followed by *Scheffe's posthoc test*[21] also revealed a different trend. The overall significance of the data was acceptable at the 0.01 level of confidence (F = 330.75, *P* < 0.01). This result indicates that there must be significant difference between the groups and within the groups including their interactions. The result also reveals that there is a significant difference between the control and experimental groups in almost all the variables (F = 11.90, *P* < 0.05). The value of interaction was also statistically significant (F = 10.94, *P* < 0.05). Thus, it can be interpreted that the mean changes in SOD, glycosylated hemoglobin and blood glucose among diabetics were statistically significant.

**Figure 1:**
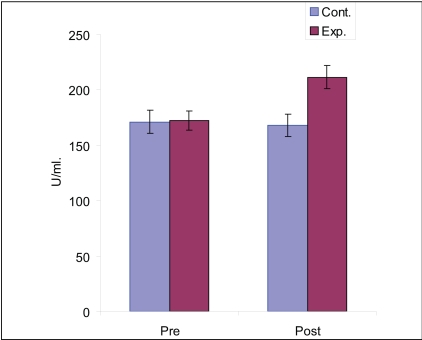
Influence of yoga training on superoxide dismutase

Further, the results of mean achievement in SOD as obtained from Scheffe's Post Hoc test [Table 2] revealed that Control group did not show any significant improvement (CD = 0.24, *P* > 0.05) [Figure 1], whereas the Experimental group showed favorable result in improving SOD significantly (CD = 0.96, *P* < 0.01). However, the overall result indicates that the experimental group had higher SOD values than the control group (CD = 0.81, *P* < 0.01). This result shows that yoga training was helpful in increasing the SOD level among diabetics.

**Table 2:** Scheffe's posthoc test for differences between pairs of ordered means in SOD activity and glycosylated Hb and blood glucose levels

Variables	(Steps)	3	2	1
SOD (Super Oxide Dismutase)	4	0.96**	0.81**	0.91**
	3	–	0.12	0.10
	2		–	0.24
	1			–
G.Hb (Glycosylated Hemoglobin)	4	0.49*	0.55**	0.58**
	3	–	0.14	0.11
	2		–	0.16
	1			–
BG (Blood Glucose)	4	0.47*	0.59**	0.63**
	3	–	0.09	0.13
	2		–	0.18
	1			–

** *P* < 0.01

* *P* > 0.05 (Where for control gr.: 1=Pretest, 2=Post test; for experimental gr.: 3=Pretest; 4=Posttest)

**Figure 2:**
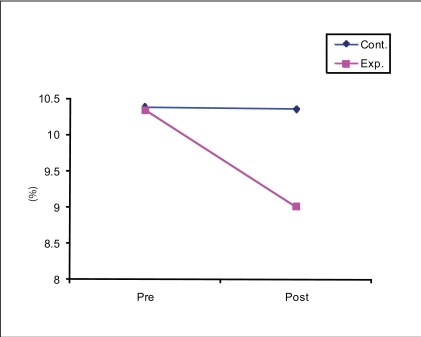
Reduction of glycosylated hemoglobin through yoga training

The impact of yoga training was different in the case of glycosylated Hb. It is interesting to note that the experimental group showed statistically significant reduction (CD = 0.49, *P* < 0.05) in glycosylated Hb levels whereas the control group did not (CD = 0.16, *P* > 0.05). Comparative results indicate that the experimental group subjects could significantly reduce their levels of glycosylated Hb as compared to the control subjects (CD = 0.55, *P* < 0.05). Thus, the six week-long Yoga training intervention was found to be useful for the reduction of glycosylated Hb levels among diabetics [Figure 2].

Blood Glucose levels are found to be generally inconsistent among diabetics. Even though both the experimental and control groups were taking allopathic medication, the experimental group also underwent the Yoga training. The result revealed that after the Yoga intervention in the experimental subjects, the levels of Blood Glucose were reduced (but within the normal range) in the experimental group (CD = 0.47, *P* < 0.05) whereas this reduction was not statistically significant in the case of the control group (CD = 0.18, *P* > 0.05). A comparison between the groups indicates that the degree of reduction of Blood Glucose was higher in the experimental subjects than in the controlled ones (CD = 0.59, *P* < 0.01). This result supports the premise that Yoga training intervention could provide an additional advantage along with medication for Blood Glucose reduction among diabetics. Thus, the simultaneous practice of Yoga with medication is perhaps, beneficial for diabetes in reducing excessive Blood Glucose.

## DISCUSSION

Diabetes is a syndrome characterized by a perturbation of glucose homeostasis and is one of the major causes of mortality worldwide. This is not only because of its ostensibly altered glucose metabolism but also due to its related secondary complications like atherosclerosis, hypertension, cerebrovascular accidents (stroke) and other associated microvascular changes leading to retinopathy, nephropathy and neuropathy due to increased oxidative stress.[22] Increased free radical formation as well as its consequent oxidative stress are seen to be characteristic features of diabetes. The mechanism underlying the apparent increased oxidative stress is not entirely clear. Accumulating evidence points to many, often-interrelated mechanisms[4,5] which may be due to autooxidation of glucose, increased generation of non-enzymatically glycosylated proteins and elevated activity of the mitochondrial electron transport chain due to an increased glucose load.[8,23] It is also known that the antioxidant defense system is depleted and that the activity of antioxidant enzymes is reduced in diabetics.[9] The role of oxidant stress in the causation of chronic tissue damage in diabetics is being increasingly recognized. Oxidant stress is usually countered by an abundant supply of antioxidants. If concomitant antioxidant deficiency occurs, oxidant stress may produce tissue damage.

In the present study, diabetic subjects were selected to monitor and evaluate the effects of a specific set of yogic exercises on one of the antioxidant enzymes, i.e., superoxide dismutase (SOD) levels along with a comparative assessment of the glycemic index in terms of glycosylated Hb and fasting blood glucose levels. *Pranayama* (breathing exercise) is documented to produce an intense calming effect on the mind and is responsible to relieve psychological stress,[24,25] which might have enhanced led to the chain of reactions relieving oxidative stress. One study conceded that there are reports showing considerable changes in the concentrations of plasma ‘catecholamines’ which in turn are correlated to changes in nasal cycles[26] as in *Anulom-Vilom Pranayama*. Catecholamines are hormones that are secreted after stimulation of sympathetic nervous impulses and which regulate diverse physiological functions in the body like the release of glucose and lipids, constriction of blood vessels, increase in blood pressure, heart rate and verve of heart contractions. They even induce blood coagulation, bronchodilatation and increase the basal metabolic rate of the body (BMR). A rise in catecholamine levels has been shown to correlate with the stimuli of psychological origins as well as other physical stimuli such as pain, injuries, trauma, heat, cold, fear, anger and various other emotional stresses. Hence in this study, it can be articulated that with the aid of correctly practised *Pranayama* techniques (included in the Yogic Exercise schedule), one might be able to induce these stress busters (catecholamines) and combat the above-cited factors effectively through negatively stimulation of the sympathetic nervous system via changes in nasal cycles.

The autonomic responses to breath-holding in *Pranayama* are altered most likely by increased vagal tone and decreased sympathetic discharges—all these mediated by altered breathing patterns. Additionally, with *Pranayama*, we can also influence the parasympathetic nervous system to lower the heart rate and blood pressure.[27,28] Several experiments carried out on animals have demonstrated increased free radical production and its augmented oxidative stress after physical and emotional stress.[29,30] With all this data from previous studies being consistent to the findings of this present study, we can state that due to Yogic exercise intervention in diabetics, the antioxidant status is enhanced along with a decrease in glycosylation Hb and blood glucose levels. Earlier studies have shown that oxidative stress is aggravated in diabetes due to decreased antioxidant enzyme levels, increased autooxidation of glucose and increased generation of non-enzymatically glycosylated proteins along with significantly elevated activity of the mitochondrial electron transport chain due to the increased glucose load.[8,9,22] Thus, the intervention of Yogic exercises perhaps, helped the diabetics to reduce the aggravation of oxidative stress by altering all the associated biochemical states.

Intriguingly enough, the findings of this study seem to be one of the significant observations in recent times in that we have shown enhanced SOD levels with decrease in glycosylated Hb and glucose levels. This may spawn a new area of research where we can draw a correlation between increased oxidative stress and increased glycosylation product levels in diabetes. This particular observation if studied further, may give us an idea as to whether increased glycosylation in diabetes is indeed a result of high levels of oxidative stress that is usually the hallmark of the disease or vice versa, i.e, whether increased glycosylation aggravates oxidative stress.
